# Can large language models serve as consultants for forensic cause of death analysis? A multidimensional evaluation

**DOI:** 10.3389/frai.2026.1752043

**Published:** 2026-07-28

**Authors:** Enhao Fu, Haojie Qin, Zhiling Tian, Hewen Dong, Donghua Zou, Xiaotian Yu, Ningguo Liu

**Affiliations:** 1 Academy of Forensic Science, Shanghai Key Laboratory of Forensic Medicine, Shanghai Forensic Service Platform, Key Laboratory of Forensic Medicine, Ministry of Justice, Shanghai, China; 2 School of Basic Medicine and Forensic Medicine, Institute of Medical Aspects of Specific Environments, Henan University of Science and Technology, Forensic Medicine Identification Center, Luoyang, Henan, China

**Keywords:** cause of death, decision support, forensic science, LLM, local deployment

## Abstract

**Introduction:**

Large language models (LLMs) have been proposed as decision support tools in medicine, yet their role in forensic cause of death analysis remains unexplored.

**Methods:**

In this study, we used 118 real-world cases spanning diverse categories of death to systematically evaluate the performance of four representative LLMs (GPT-4o, OpenAI o3, Gemini-2.5pro, and DeepSeek-R1) in forensic cause of death analysis. Two senior forensic pathologists independently evaluated each model’s decision-making capabilities regarding inference quality and conclusion accuracy. These metrics were assessed using an expert scoring system with a 5-point Likert scale, with original analytical statements and legally valid expert opinions serving as objective gold standards. In a sub-study, we examined the application potential of the locally deployed open-source model DeepSeek-R1:32b. Additionally, a targeted retrospective analysis was conducted to quantify the incidence and typologies of AI hallucinations.

**Results:**

DeepSeek-R1 demonstrated a statistically significant advantage in inference quality scores over GPT-4o (*p* = 0.015, *r*_rb_ = 0.28) and Gemini-2.5pro (*p* = 0.000003, *r*_rb_ = 0.46), while no statistically significant differences were observed among the four models in terms of conclusion accuracy scores. The locally deployed DeepSeek-R1:32b model also showed no statistically significant difference from GPT-4o in conclusion accuracy scores. However, hallucinations persistently appear in the response reports of all LLMs.

**Discussion:**

LLMs can provide limited auxiliary value in cause of death analysis but should not replace the final judgment of forensic experts. LLMs still require expert oversight to ensure evidence integrity and mitigate risks such as hallucination. Open source LLMs can further mitigate data privacy concerns and provide practical support for cause of death analysis.

## Highlights

1. Evaluating large language models’ ability to determine cause of death using real forensic cases.
2. Among tested large language models, DeepSeek R1 demonstrates statistically significant advantages in inference quality.
3. The locally deployed, open-source model DeepSeek-R1:32b achieves conclusion accuracy comparable to GPT-4 while ensuring data privacy.
4. Large language models can assist forensic work, but require expert human oversight.

## 1 Introduction

Cause of death analysis is a critical component of public health governance, holding significant importance for public health security, disease surveillance, and policy formulation (Patel et al., 2025). When an individual died due to illness or natural causes, the determination of the cause of death was primarily carried out by clinicians, who issued medical death certificates based on past medical history, treatment records, and clinical manifestations (Inoue et al., 2023). However, when dealing with unnatural deaths or complex circumstances that cannot be fully explained by medical causes, forensic experts must intervene. They must engage in causal inference and comprehensive judgment within complex, fragmented evidence to determine the cause of death (Madea et al., 2022). Traditional methods of determining the cause of death often rely on the knowledge and experience of individual forensic experts, resulting in issues such as high subjectivity and insufficient consistency across cases (Meilia et al., 2020). Moreover, the conclusions of cause of death analysis not only serve medical or forensic fields but are also relied upon by non-specialist judicial groups such as judges, prosecutors, law enforcement officers, and attorneys. These users, lacking systematic forensic backgrounds, are often more susceptible to information biases when interpreting or applying such conclusions, thereby introducing potential judicial risks (Obafunwa et al., 2018; Scott, 2017). Therefore, how to leverage emerging technologies to enhance the objectivity and reproducibility of cause of death analysis, while ensuring that conclusions remain transparent and reliable for diverse user groups, represents a major challenge we must address today.

The rapid advancement of Large Language Models (LLMs) presents new opportunities for addressing this issue. Since OpenAI launched ChatGPT in 2022 (Mesko, 2023), the application scope of LLMs has gradually shifted from simple text generation to more sensitive information extraction and reasoning tasks (Feuerriegel et al., 2024). In recent years, the integration of LLMs with decision-making systems has emerged as a promising research direction (Kim et al., 2025; Cho et al., 2024). Research has shown that ChatGPT can not only be used in forensic practice for decision-making support tasks, such as simulating events and predicting outcomes (Dinis-Oliveira and Azevedo, 2023), but also assist forensic professionals in drafting forensic reports (Michelet and Breitinger, 2024). Moreover, researchers have demonstrated the potential of LLMs in digital forensics applications (Wickramasekara et al., 2025). Of course, as LLMs become increasingly prevalent within the judicial system, their application will extend beyond forensic professionals to include judicial practitioners without a forensic background. Under these circumstances, it becomes more difficult to accurately assess the logical soundness of the model’s outputs and the reliability of the evidence. However, there remains a lack of systematic assessment of LLMs’ inference capabilities for “decision-making tasks” in the field of forensic science. Particularly concerning the core forensic task of determining the cause of death, the consistency of LLMs’ inference, the interpretability of results, and their ability to generalize across cases have yet to be thoroughly explored. More critically, existing research largely relies on closed-source models hosted in the cloud. Given the highly sensitive and confidential nature of forensic data, ensuring data security and privacy while maintaining model availability remains a paramount concern (Tyagi et al., 2025).

In recent years, several new popular models have gradually attracted widespread research interest (Chiang et al., 2024). In benchmark testing on Lmarena.ai, models such as ChatGPT, Gemini, and DeepSeek demonstrated significant reasoning potential (Arena Leaderboard, 2025), exhibiting strong generalization capabilities and applicability in the medical field (Sandmann et al., 2024; Sandmann et al., 2025). Among them, ChatGPT is the LLM that has been studied most extensively to date in fields such as education, healthcare, and medicine (Cascella et al., 2023; Cooper, 2023). OpenAI has released its latest LLMs, including ChatGPT-4.5, ChatGPT-4o, and OpenAI o3, allowing professionals to select different models based on their specific needs (OpenAI research, 2025). Moreover, the Gemini series of LLMs developed by Google demonstrates exceptional strengths in long-context processing, making it exceptionally well-suited for forensic reasoning and decision-making tasks that involve vast amounts of medical and autopsy textual data. The newly launched Gemini-2.5pro has swiftly claimed the top position on the Lmarena.ai leaderboard (Google, 2025). DeepSeek, as an open-source LLM developed in China, sparked extensive academic discussion upon its release (Gibney, 2025). This technology enables researchers and forensic professionals to optimize models according to specific requirements, allowing forensic institutions or individuals to deploy them locally for operation. This provides additional privacy and security safeguards for sensitive medical data and forensic documents (Temsah et al., 2025).

Based on the above background, we selected three closed-source models: GPT-4o, OpenAI o3, and Gemini-2.5pro, along with one open-source model, DeepSeek-R1, to evaluate the decision support capabilities of LLMs in forensic cause-of-death analysis tasks. By constructing a standardized forensic reasoning task framework, we compare its performance across dimensions such as inference quality and conclusion accuracy. Additionally, we further examined the applicability of each model across different case types and tentatively explored the potential of open-source LLM (DeepSeek-R1:32b) under localized deployment conditions. We aim to provide a systematic and actionable reference for the practical application of LLMs in the field of forensic science and to establish an empirical foundation for the future development of judicial artificial intelligence.

## 2 Materials and methods

**Figure 1:**
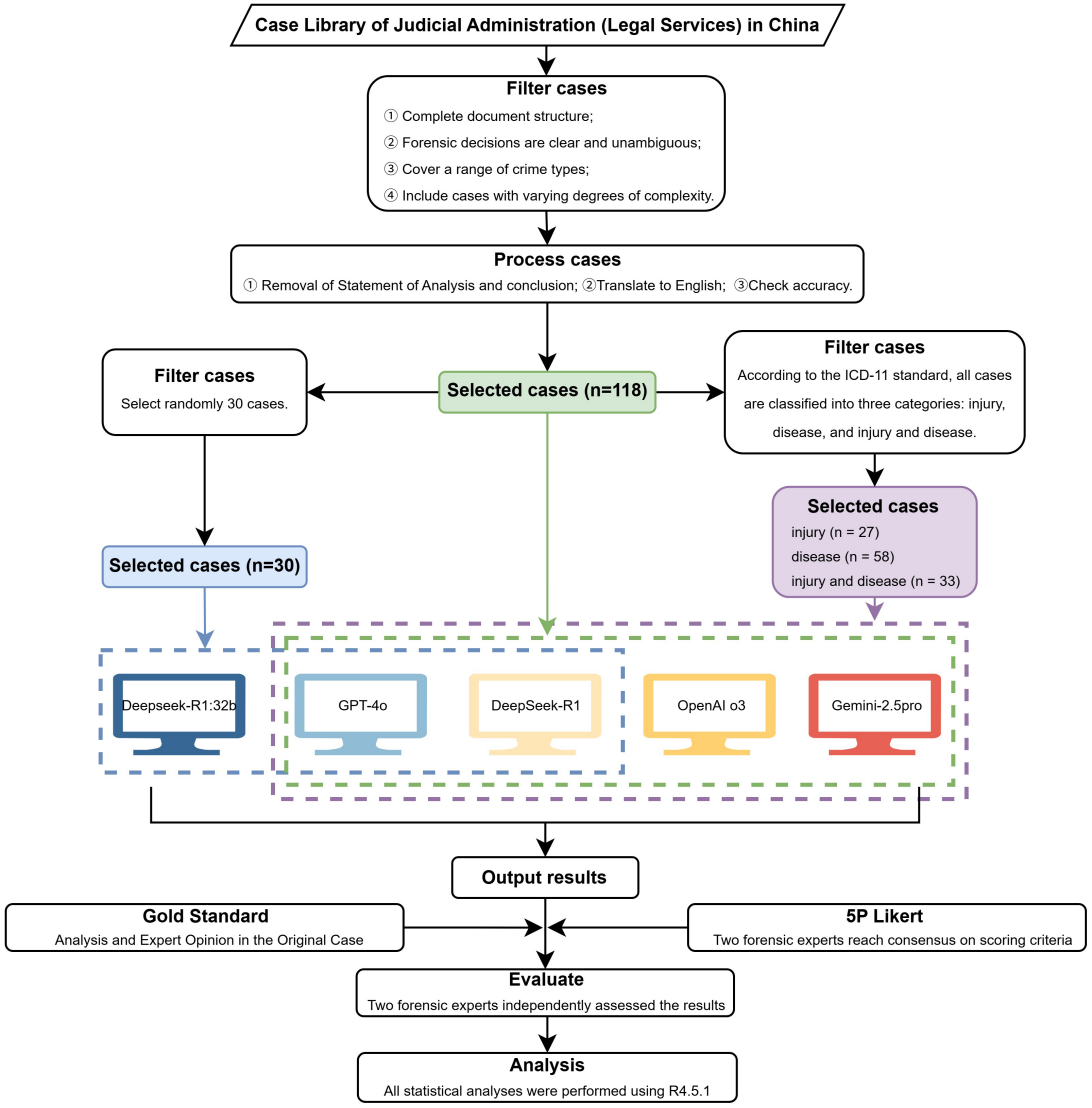
Workflow overview.

Our workflow for selecting forensic case reports, querying LLMs, and evaluating results is shown in Figure 1.

### 2.1 Data source

In order to ensure that the research is grounded in principles of representative and impartial case selection, all cases utilized in this study have been sourced from the *Case Library of Judicial Administration (Legal Services) in Public Legal Services of China* (Case Library of Judicial Administration (Legal Services), 2025). This case repository is published by official government authorities. The cases included have been reviewed and endorsed by authoritative experts appointed by the government, serving as a reference guide for forensic identification nationwide. Forensic experts reviewed all case data from the national-level identification center within the case repository, ultimately selecting 118 cases as test data for further analysis (Supplementary Data 1). The selection criteria are as follows: (1) the case should contain a complete document structure, (2) cases should cover a range of crime types, such as sudden death, tangle, medical, traffic accidents, etc. (3) include cases with varying degrees of complexity, from relatively straightforward and direct cases to more intricate ones involving multiple suspects, diverse evidence types, and complex timelines. Steps 1 is essential, as they form the prerequisite for our decision-making tasks. Steps 3 and 4 are designed to enrich the diversity and complexity of the cases, thereby further systematically testing the performance of the LLMs. Because the cases are all drawn from real events, there is no overly idealized or ambiguous evidence, ensuring the quality of our test data. Furthermore, as these are publicly available cases from the *Public Legal Services of China*, the case data does not raise ethical concerns or privacy breaches.

To generate the final decision-making problems for testing, we removed the analytical statements and identification conclusions from each case. Since most of the large models tested use English data as their primary training corpus, to reduce the risk of decision bias arising from language differences, we used the translation tool DeepL^1^ to translate the cases included in the study into English. Ahuja et al.’s research also shows that models consume more tokens when processing non-English content, resulting in inefficiency and even miscommunication. Compared to asking questions directly in one’s native language, translating non-English input into English can lead to significant performance improvements in complex reasoning tasks (Ahuja et al., 2023). We also conducted a manual review of the translated case studies to ensure linguistic quality and accuracy.

### 2.2 Public LLM access

In this study, we used four AI-based chatbots via the official website: ChatGPT-4o,^2^ OpenAI o3,^3^ Gemini-2.5pro,^4^ and DeepSeek-R1.^5^

To ensure the reproducibility of the experiment, we adopted a zero-shot prompting strategy (Yu et al., 2025). Prompts are constructed using the following components: “role definition” + “zero-shot instructions” + “output constraints” + “format requirements.” Ultimately, all questions were answered using a standardized prompt, which was “You are an experienced medical examiner. Based on the case information provided below, please conduct a professional and rigorous cause-of-death analysis and produce a concise report containing both a “Statement of Analysis” and a “Conclusion” in the format of a formal coroner’s report. This standardized zero-shot approach is utilized to assess the models’ inherent reasoning capabilities independently of complex prompt engineering. Although the instructions are simple, the case data provided follows a structured forensic workflow, and the clearly defined roles and output scope enable the LLM to generate high-quality reasoning results (Wang et al., 2024).

In addition, to ensure strict experimental control and prevent information leakage, a completely new and independent chat session was initiated for all four LLMs for each evaluated case. To reflect the benchmark performance of these publicly available large language models, specifically their inference performance under default configurations, the original default model parameter settings (e.g., temperature and maximum token limits) were maintained across all platforms without manual intervention. All question-and-answer tests were completed between June 5 and 15, 2025.

### 2.3 Exploring the performance of LLMs in different types of cases

According to the classification principles of the *International Classification of Diseases, 11th Revision* (ICD-11) (ICD-11 for Mortality and Morbidity Statistics, 2025), all 118 cases in this study were systematically categorized into three groups for the purpose of statistical analysis: (i) injury, (ii) disease, (iii) injury combined with disease.

### 2.4 Exploratory research on local open-source LLM

To further explore the applicability and efficacy of locally deployed open-source LLMs in forensic practice, we deployed the DeepSeek-R1:32b model in a local environment using the Ollama framework (Ollama, 2025). To fully validate the performance of analyzing locally deployed open-source LLMs, we selected DeepSeek-R1 and GPT-4o as comparison models for systematic testing. Because the DeepSeek-R1 versus DeepSeek-R1:32b test group can validate the impact of reduced model parameters on inference performance, while the GPT-4o versus DeepSeek-R1:32b test group can validate the potential of local open-source LLMs to replace closed-source LLMs.

We randomly selected 30 cases from the 118 cases mentioned above to form a sample group for testing. To ensure the statistical validity and generalizability of this sample, we conducted a representativeness test and used Fisher’s exact test to compare the categories of cause of death between the selected 30 cases and the overall dataset (*p* = 0.747). All questions were answered using the same prompts as the publicly available LLMs. Each generated output queried using DeepSeek-R1:32b is provided in Supplementary Data 2.

### 2.5 Performance evaluation

To ensure the validity and objectivity of the evaluation, all output generated by GPT-4o, OpenAI o3, Gemini-2.5pro, DeepSeek-R1, and the local DeepSeek-R1:32b model was anonymized and randomized so that evaluators could not identify the source model. Two professional forensic experts assessed content generated. Both experts hold senior forensic pathology titles (associate chief physician or above) and have over 15 years of experience in forensic pathology and judicial identification. To minimize subjective bias in the evaluation process, expert assessments are based on the legally binding analytical conclusions and expert opinions contained in the original documentation as objective reference benchmarks. The evaluation of the model focuses on two distinct dimensions: inference quality and conclusion accuracy. Two forensic pathologists independently assessed five randomly selected cases and reached consensus on the final Likert scale criteria (the scoring criteria are provided in Supplementary Table 2). Each forensic pathologist then rated the model’s decision-making inference using a 5-point Likert scale (1 = very poor, 5 = excellent). The final score was calculated as the average of the two individual ratings.

All statistical analyses were conducted using R 4.5.1. To assess inter-rater reliability, we used the R package DescTools 0.99.60 to calculate the weighted Cohen’s kappa (function “cohenkappa,” weights “Equal-spacing”) and 95% confidence intervals (CIs) for each of the two tasks. The mean of the two raters’ scores was utilized for subsequent comparative analyses only after this baseline agreement was established. To determine whether there are overall performance differences among the general-purpose LLM models GPT-4o, OpenAI o3, DeepSeek-R1, and Gemini-2.5pro, we first conducted a Friedman test. If the Friedman test results were significant, we performed further analysis using the Wilcoxon signed-rank test (R package base, function wilcox.test with argument paired = TRUE). The performance of GPT-4o, OpenAI o3, DeepSeek-R1, and Gemini-2.5pro across different case types was similarly analyzed using the aforementioned testing strategies. For the locally developed, open-source LLM, we set up two groups of additional comparative analyses. The performance differences between DeepSeek-R1:32b and both DeepSeek-R1 and GPT-4o were analyzed for significance using a one-sided Wilcoxon signed-rank test. Finally, all multiple tests were adjusted using Bonferroni correction (Bonferroni, 1936), and statistical significance was defined as *p* < 0.05. Effect sizes for these comparisons were calculated and reported as the rank-biserial correlation (*r*_rb_).

### 2.6 Hallucination evaluation

In addition to overall performance evaluation metrics, we conducted a targeted retrospective analysis aimed at quantifying the occurrence of hallucinations in reports generated by LLM. In this study, we draw upon existing methodologies to classify hallucinations into six distinct types (Table 1) (Massenon et al., 2025). Two forensic experts independently reviewed all model outputs, coding the presence of each hallucination type within a given case as a binary variable (Yes/No). Any discrepancies between the evaluators were resolved through consensus discussion.

**Table 1:** Taxonomy of LLM hallucinations.

Hallucination type	Definition	Example
Factual Incorrectness	The information provided by the LLM is verifiably false or contradicts established facts within the forensic field or common sense.	The weight of the organ listed does not match the actual weight.
Fabricated Information/Invention	LLM generates details, entities, features, or sources that are entirely non-existent or not present within the app’s context or reality.	The identification materials did not mention toxicological testing, but this section appeared in the analytical notes.
Nonsensical/Irrelevant Output	LLM produces responses that are grammatically sound but semantically meaningless, incoherent, repetitive, or completely off-topic to the user’s query or interaction.	The input content pertains to cases of death at the scene, but the model is prompting for responsibilities to be addressed during the medical process.
Logical Inconsistency/Self-Contradiction	LLM’s output contains statements that contradict each other within the same response, across a short conversational turn, or demonstrates clearly flawed reasoning.	The model initially mentioned traumatic brain injury, but in the summary, it changed to “no significant abnormalities were found in the cranial examination.”
Persona Deviation/Role Inconsistency	The AI’s responses deviate significantly from its established persona, intended role within the app, or the expected tone, potentially using inappropriate or unexpected language.	In the preparation of what should be a solemn forensic autopsy report, highly unprofessional colloquial, emotional, and even anthropomorphic expressions were employed.
Repetitive Output (Non-functional)	LLM gets stuck in a loop, repeating the same phrase, sentence, or set of characters nonsensically and without progression, often indicating a failure state.	The model stalled while analyzing the fatal pathology chain and began endlessly repeating the same phrase.

## 3 Results

### 3.1 Inter-rater reliability

Inter-rater reliability was determined using weighted Cohen’s *κ* (Table 2). In the inference quality task, GPT-4o achieved κ = 0.52, OpenAI o3 achieved κ = 0.58, Gemini-2.5pro achieved κ = 0.50, DeepSeek-R1 achieved κ = 0.50, and DeepSeek-R1:32b achieved κ = 0.72. In the conclusion accuracy task, GPT-4o achieved a κ = 0.72, OpenAI o3 achieved a κ = 0.74, Gemini-2.5pro achieved a κ = 0.67, DeepSeek-R1 achieved a κ = 0.82, and DeepSeek-R1:32b achieved a κ = 0.71. According to Landis and Koch (1977), inter-rater reliability levels can be interpreted as ranging from moderate (0.41–0.60) to high (0.61–0.80). Notably, in the task of conclusion accuracy, consistency was significantly higher. This result indicates that the scoring scheme employed in this study exhibits acceptable inter-rater reliability when assessing the inference quality and conclusion accuracy of various LLMs, thereby supporting the reliability of the scoring outcomes.

**Table 2:** Inter-rater reliability considering a 5-point Likert scale.

Models	Inference quality		Conclusion accuracy	
	Cohen’s κ	95% CI	Cohen’s *κ*	95% CI
GPT-4o	0.52	[0.39, 0.65]	0.72	[0.62, 0.81]
OpenAI o3	0.58	[0.46, 0.70]	0.74	[0.65, 0.83]
Gemini-2.5pro	0.50	[0.36, 0.63]	0.67	[0.57, 0.79]
DeepSeek-R1	0.50	[0.44, 0.67]	0.82	[0.74, 0.90]
DeepSeek-R1:32b	0.72	[0.51, 0.94]	0.71	[0.53, 0.90]

### 3.2 Model performance for inference quality task

**Figure 2:**
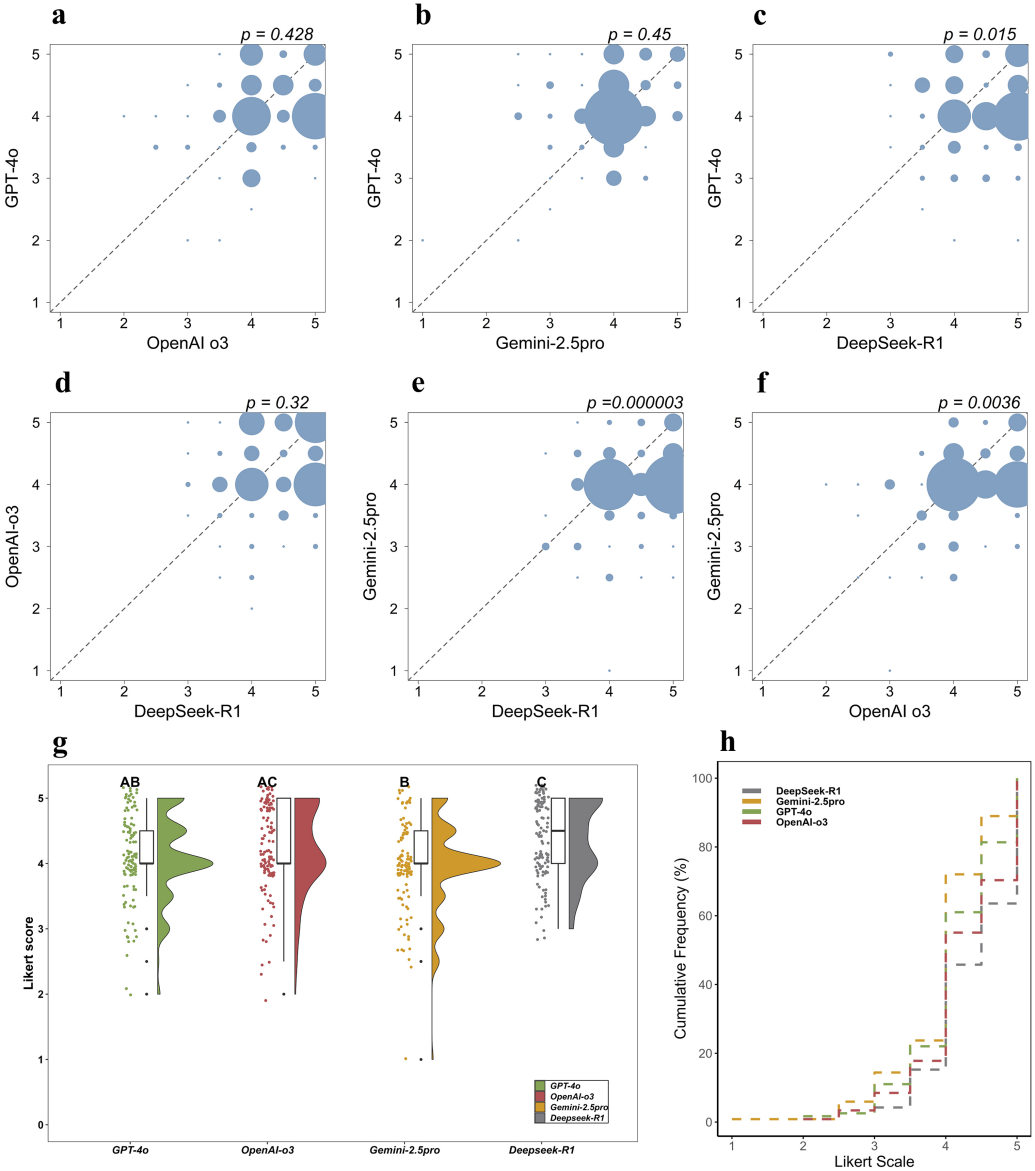
Model performance for the inference quality task. **(a–f)** Bubble plots showing the results of the 118 pairwise comparison inference quality between four models: GPT-4o, OpenAI o3, DeepSeek-R1, and Gemini-2.5pro. **(a)** OpenAI o3 versus GPT-4o (two-sided Wilcoxon signed-rank test with continuity correction, Bonferroni correction with k = 6, adjusted *p* = 0.428, V = 2180.5, 95% CI −6.78 × 10–6 to 0.25, estimate 0.25); **(b)** Gemini-2.5pro versus GPT-4o (two-sided Wilcoxon signed-rank test with continuity correction, Bonferroni correction with *k* = 6, adjusted *p* = 0.45, V = 1,327, 95% CI −0.5 to 3.28 × 10–5, estimate −0.25); **(c)** DeepSeek-R1 versus GPT-4o (two-sided Wilcoxon signed-rank test with continuity correction, Bonferroni correction with k = 6, adjusted *p* = 0.015, V = 2,842, 95% CI 1.95 × 10–5 to 0.75, estimate 0.25); **(d)** DeepSeek-R1 versus OpenAI o3 (two-sided Wilcoxon signed-rank test with continuity correction, Bonferroni correction with k = 6, adjusted *p* = 0.32, V = 2210.5, 95% CI −6.91 × 10–5 to 0.25, estimate 0.25); **(e)** DeepSeek-R1 versus Gemini-2.5pro (two-sided Wilcoxon signed-rank test with continuity correction, Bonferroni correction with k = 6, adjusted *p* = 0.000003, V = 2,954, 95% CI 0.50 to 0.75, estimate 0.75); **(f)** OpenAI o3 versus Gemini-2.5pro (two-sided Wilcoxon signed-rank test with continuity correction, Bonferroni correction with k = 6, adjusted *p* = 0.0036, V = 2375.5, 95% CI 0.25 to 0.75, estimate 0.50); **(g)** Raincloud plot shows the Likert scale score distribution of inference quality performance differences between GPT-4o, OpenAI o3, DeepSeek-R1, and Gemini-2.5pro. The violin plot shows the score distribution, with embedded boxplots indicating the median and interquartile range, and scatter points representing each case’s score. Uppercase letters denote CLD results, with no significant differences between groups sharing the same letter (*α* = 0.05); **(h)** The cumulative frequency of Likert scale scores for inference quality for GPT-4o, OpenAI o3, DeepSeek-R1, and Gemini-2.5pro.

For the inference quality task within the forensic decision evaluation framework, Friedman test results indicate significant performance disparities exist among general-purpose LLMs (X^2^ = 27.163, *p <* 0.001; detailed data is provided in Supplementary Table 1). DeepSeek-R1 significantly outperforming GPT-4o (*p* = 0.015, *r*_rb_ = 0.28) and Gemini-2.5pro (*p* = 0.000003, *r*_rb_ = 0.46). We did not observe any performance difference between OpenAI o3 and GPT-4o, but OpenAI o3 showed a statistically significant difference compared to Gemini-2.5pro (*p* = 0.0036, *r*_rb_ = 0.32). Figure 2 visualizes the results of pairwise comparisons (bubble plots) of the inference quality among GPT-4o, OpenAI o3, DeepSeek-R1, and Gemini-2.5pro, along with performance comparisons (raincloud plot and cumulative frequency plot) between the four models.

### 3.3 Model performance for the conclusion accuracy task

**Figure 3:**
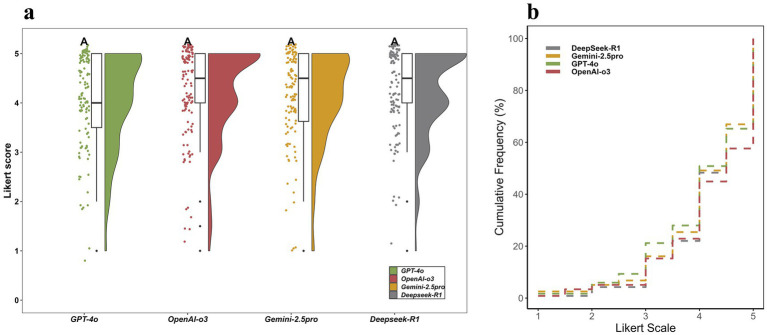
Model performance for the conclusion accuracy task. **(a)** Raincloud plot shows the Likert scale score distribution of conclusion accuracy performance differences between GPT-4o, OpenAI o3, DeepSeek-R1, and Gemini-2.5pro. The violin plot shows the score distribution, with embedded boxplots indicating the median and interquartile range, and scatter points representing each case’s score. Uppercase letters denote CLD results, with no significant differences between groups sharing the same letter (α = 0.05); **(b)** the cumulative frequency of Likert scale scores for conclusion accuracy for GPT-4o, OpenAI o3, DeepSeek-R1, and Gemini-2.5pro.

In the task of evaluating the accuracy of LLM conclusions, Friedman test results indicate no statistically significant differences were found among the four LLMs (X^2^ = 3.803, *p* = 0.284). Figure 3 shows the performance of the four models.

### 3.4 Performance of LLMs across different case types

**Figure 4:**
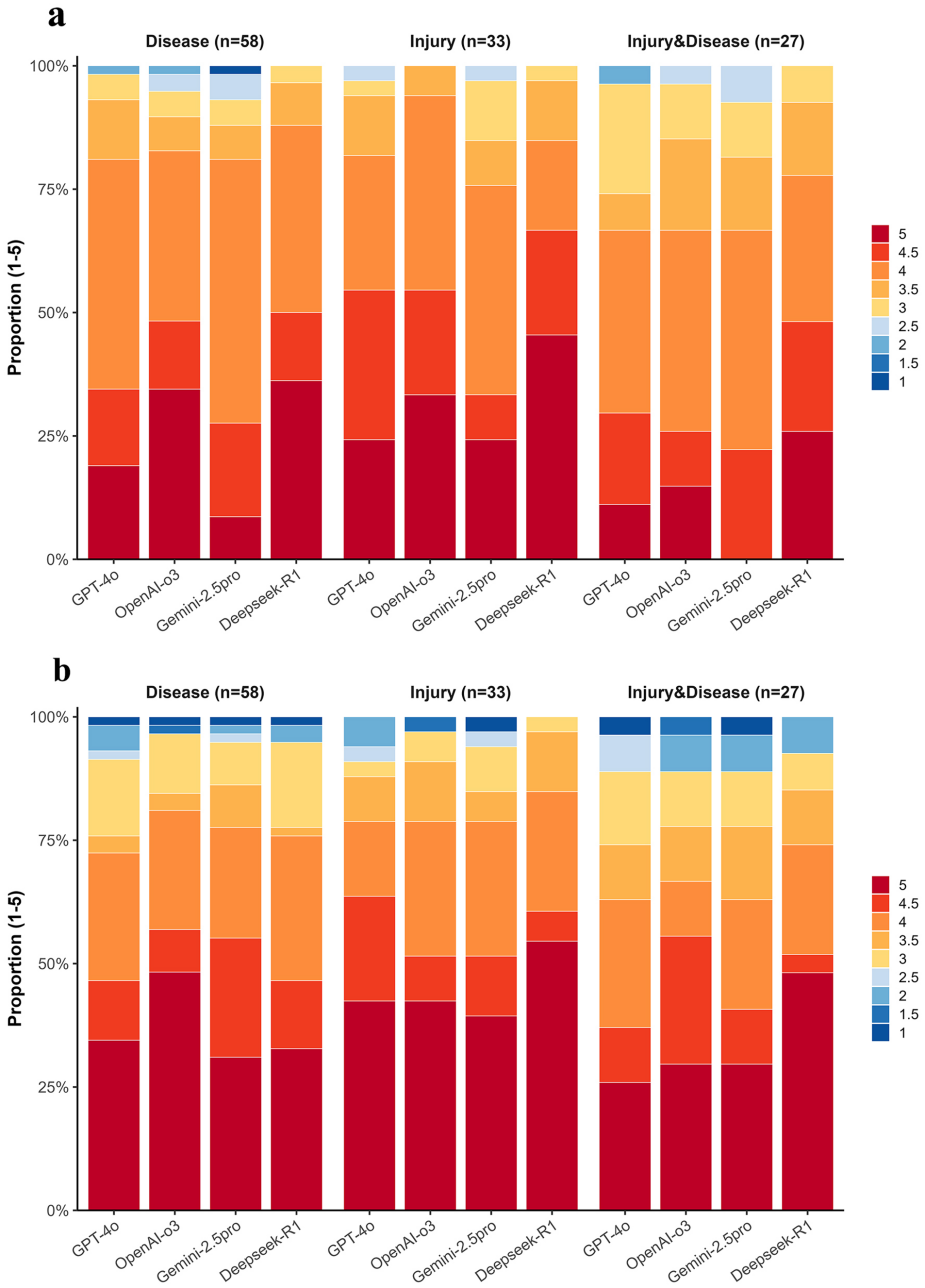
Likert 100% stacked bar plot. **(a,b)** Stacked bar plot illustrates the performance of GPT-4o, OpenAI-o3, DeepSeek-R1, and Gemini-2.5pro across disease (*n* = 58), injury (*n* = 33), and mixed disease-injury (*n* = 27) cases. Colors represent rating levels (1–5). **(a)** Scoring proportion for the inference quality; **(b)** Scoring proportion for the conclusion accuracy.

**Figure 5:**
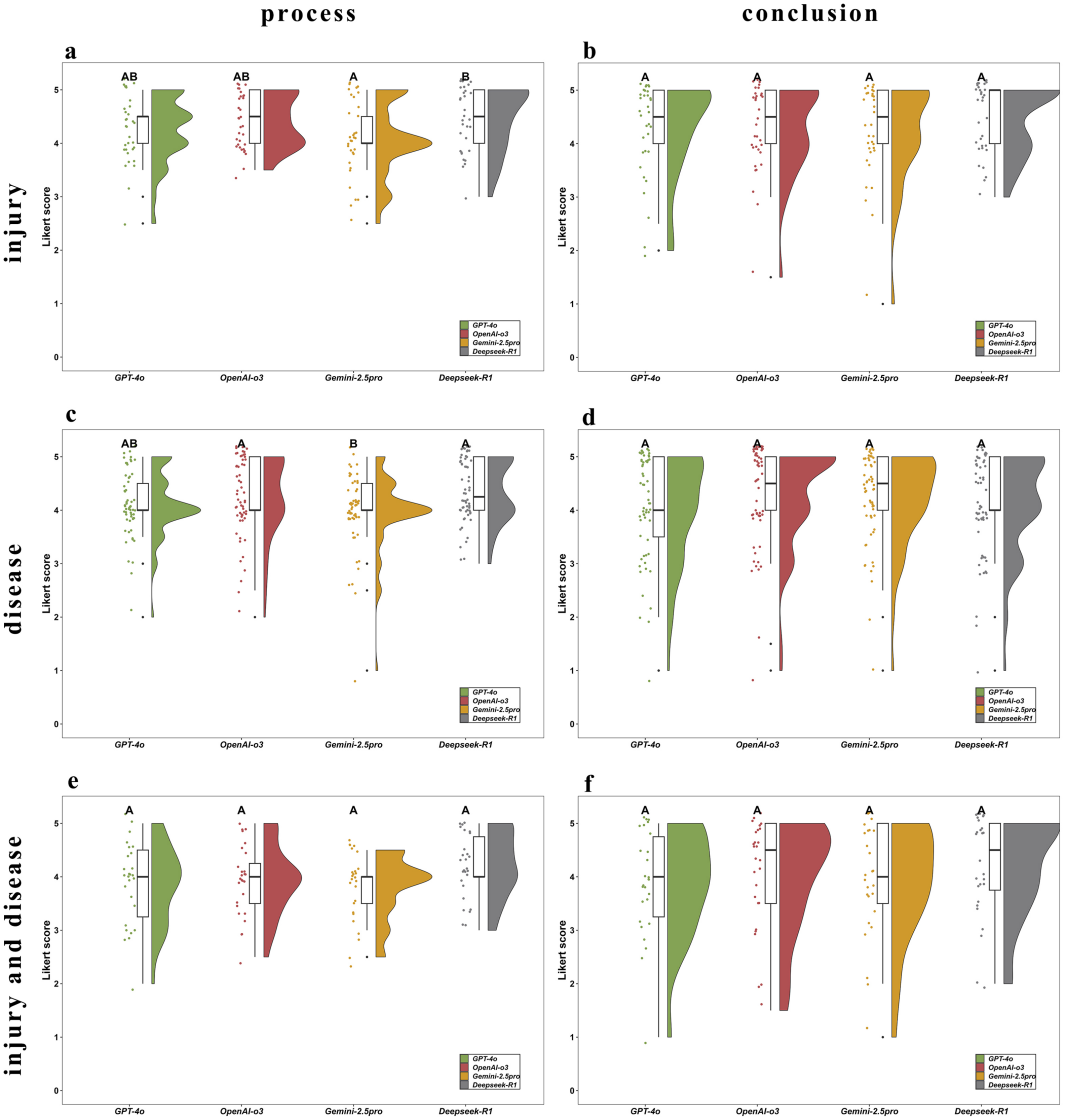
Comparative performance of LLM across different case types. Raincloud plots show the Likert scale distribution of model performance for GPT-4o, OpenAI-o3, DeepSeek-R1, and Gemini-2.5pro in injury, disease, and injury-disease cases. The violin plot shows the score distribution, with embedded boxplots indicating the median and interquartile range, and scatter points representing each case’s score. Uppercase letters denote CLD results, with no significant differences between groups sharing the same letter (*α* = 0.05). **(a,c,e)** Scoring distribution for inference quality; **(b,d,f)** Scoring distribution for conclusion accuracy.

As shown in Figure 4, the four models primarily scored between 3 and 5 points across injury, disease, and combined injury-and-disease cases. In the injury-type inference quality task (Figure 5a), DeepSeek-R1 demonstrated superior performance compared to Gemini-2.5pro (*p* = 0.016, r_rb_ = 0.52). In the disease-type inference quality task (Figure 5b), DeepSeek-R1 (*p* = 0.00012, r_rb_ = 0.49) and OpenAI o3 (*p* = 0.04, r_rb_ = 0.36) both demonstrated statistically significant differences compared to Gemini-2.5pro. It is important to note that, in all tasks of conclusion accuracy (Figures 5d,f), we did not find any performance differences among the four models across the three case types. Detailed metrics can be found as Supplementary Tables 5–7.

### 3.5 Local model testing results

**Figure 6:**
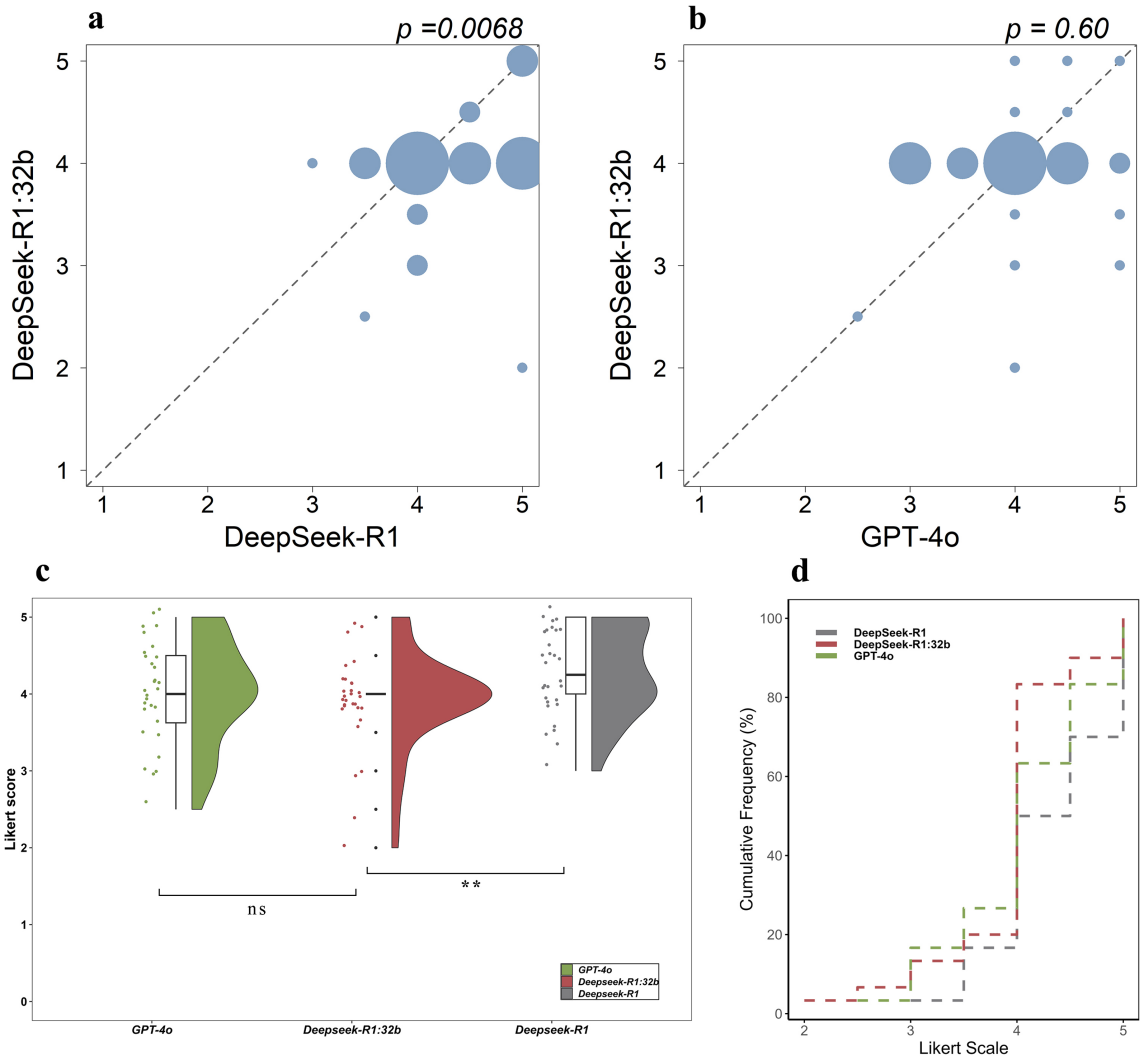
Local model performance for the inference quality task. **(a,b)** Bubble plots showing the results of 30 pairwise comparison inference quality among three models: DeepSeek-R1, GPT-4o, and DeepSeek-R1:32b. **(a)** DeepSeek-R1 versus DeepSeek-R1:32b (one-sided Wilcoxon signed-rank test with continuity correction, alternative = greater, Bonferroni correction with *k* = 2, adjusted *p* = 0.0068, V = 161.0, 95% CI 0.25 to infinity, estimate 0.75); **(b)** GPT-4o versus DeepSeek-R1:32b (one-sided Wilcoxon signed-rank test with continuity correction, alternative = greater, Bonferroni correction with *k* = 2, adjusted *p* = 0.60, V = 131, 95% CI −0.25 to infinity, estimate 7.54 × 10^−6^); **(c)** Raincloud plot shows the Likert scale score distribution of inference quality performance differences between DeepSeek-R1, GPT-4o, and DeepSeek-R1:32b. The violin plot shows the score distribution, with embedded boxplots indicating the median and interquartile range, and scatter points representing each case’s score; **(d)** the cumulative frequency of Likert scale scores for inference quality for DeepSeek-R1, GPT-4o, and DeepSeek-R1:32b.

**Figure 7:**
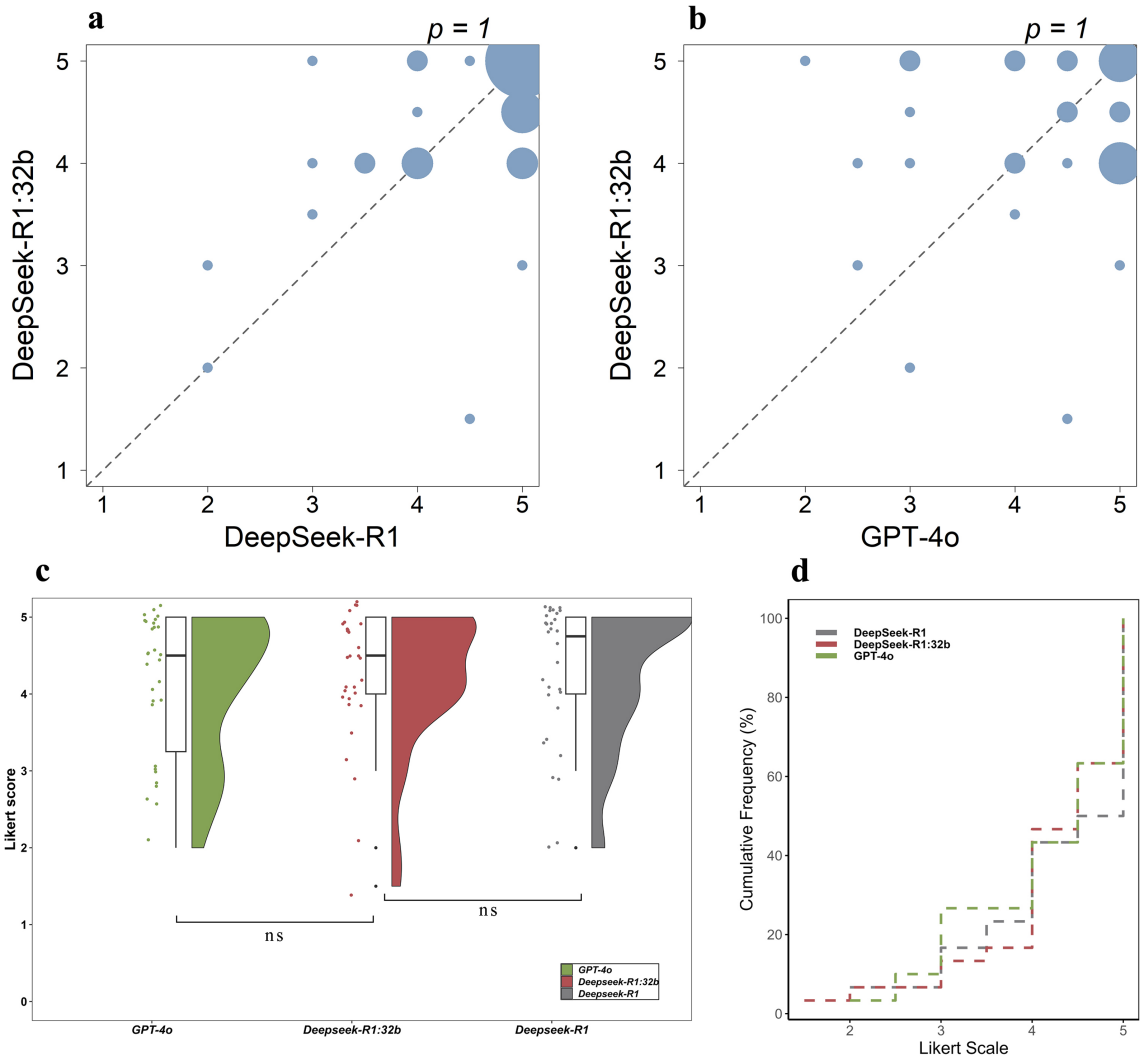
Local model performance for the conclusion accuracy task. **(a,b)** Bubble plots showing the results of 30 pairwise comparison conclusion accuracy among three models: DeepSeek-R1, GPT-4o, and DeepSeek-R1:32b. **(a)** DeepSeek-R1 versus DeepSeek-R1:32b (one-sided Wilcoxon signed-rank test with continuity correction, alternative = greater, Bonferroni correction with *k* = 2, adjusted *p* = 1 V = 95.5, 95% CI −0.5 to infinity, estimate 4.24 × 10^−5^); **(b)** GPT-4o versus DeepSeek-R1:32b (one-sided Wilcoxon signed-rank test with continuity correction, alternative = greater, Bonferroni correction with *k* = 2, adjusted *p* = 1, V = 114, 95% CI −0.75 to infinity, estimate −8.53 × 10^−5^); **(c)** Raincloud plot shows the Likert scale score distribution of conclusion accuracy performance differences between DeepSeek-R1, GPT-4o, and DeepSeek-R1:32b. The violin plot shows the score distribution, with embedded boxplots indicating the median and interquartile range, and scatter points representing each case’s score. **(d)** The cumulative frequency of Likert scale scores for conclusion accuracy for DeepSeek-R1, GPT-4o, and DeepSeek-R1:32b.

We also evaluated the performance of the local model across two dimensions: inference quality and conclusion accuracy. In the comparative test of inference quality tasks (Figure 6), DeepSeek-R1 demonstrated a significant difference compared to the local model (*p* = 0.0068, r_rb_ = 0.49), while no difference was observed between GPT-4o and 32b. Furthermore, we did not observe any statistically significant differences in the task of inference conclusion (Figure 7). Detailed metrics can be found as Supplementary Table 4.

### 3.6 Hallucination assessment results

Table 3 summarizes the occurrence rates of various hallucination types across the evaluated LLMs. Factual incorrectness is the most frequently occurring type of hallucination across all models, with incidence rates ranging from 24.8% (OpenAI o3) to 31.9% (GPT-4o). The local model, DeepSeek-R1:32b, exhibited a comparatively higher rate of fabricated information (23.3%) than the top-tier cloud-based models. All models demonstrated excellent prompt adherence, with zero instances of persona deviation recorded across the entire dataset. Furthermore, non-functional repetitive output was virtually non-existent, occurring only once (3.3%) in the local model.

**Table 3:** Incidence of hallucinations in LLM reports.

Hallucination type	GPT-4o	OpenAI o3	DeepSeek-R1	Gemini-2.5pro	DeepSeek-R1: 32b
Factual incorrectness	27 (31.9%)	21 (24.8%)	23 (27.1%)	26 (30.68%)	9 (30.0%)
Fabricated information/Invention	11 (13.0%)	9 (10.6%)	15 (17.7%)	14 (16.6%)	7 (23.3%)
Nonsensical/Irrelevant output	13 (15.3%)	8 (9.4%)	9 (10.6%)	16 (18.9%)	4 (13.3%)
Logical inconsistency/Self-contradiction	8 (9.4%)	9 (10.6%)	12 (14.2%)	13 (15.3%)	2 (6.7%)
Persona deviation/Role inconsistency	0 (0.0%)	0 (0.0%)	0 (0.0%)	0 (0.0%)	0 (0.0%)
Repetitive Output (non-functional)	0 (0.0%)	0 (0.0%)	0 (0.0%)	0 (0.0%)	1 (3.3%)

Each case is counted only once for each type of illusion, and different types of illusions do not influence each other. The percentage represents the proportion of cases containing that specific error out of the total cases (*N* = 118 or *N* = 30).

## 4 Discussion

Artificial intelligence is rapidly becoming a vital tool in the field of forensic science, particularly as LLMs like ChatGPT have demonstrated immense potential and promising applications in the medical domain (Sandmann et al., 2024). In our study, two forensic experts carefully evaluated the performance of current, general-purpose LLMs and found that they can be used to assist forensic personnel in performing the task of inferring the cause of death. Previous studies have demonstrated the potential applications of LLMs in forensic decision-making. For example, LLMs can analyze context to extract key information related to criminal situations from text messages and chat logs, providing investigators with more robust and intuitive evidence analysis data, thereby enhancing forensic analysis and criminal investigation capabilities (Kim et al., 2025). In recent research, the use of LLMs has also been shown to significantly enhance the ability to analyze complex legal and medical documents, thereby helping forensic professionals reach more scientific and informed conclusions (Petroni et al., 2025).

In this study, we focus on the performance of LLMs in two tasks: the conclusion accuracy and inference quality in forensic decision-making. We observed that GPT-4o, OpenAI o3, DeepSeek-R1, and Gemini-2.5pro all effectively completed the task of cause of death analysis and accurately adopted forensic medical terminology. In addressing inference quality tasks, compared to Gemini-2.5pro, both OpenAI o3 (*p* = 0.0036, *r*_rb_ = 0.32) and DeepSeek-R1 (*p = 0.000003, r_rb_ = 0.46*) demonstrate superior capabilities. Furthermore, DeepSeek-R1 shows notable differences when compared to GPT-4o (*p = 0.015, r_rb_ = 0.28*). However, in the task of conclusion accuracy, no statistically significant differences (Figure 3) were detected among all comparison models (GPT-4o, OpenAI o3, DeepSeek-R1, and Gemini-2.5pro).

Further analysis of model outputs shows that, in some cases, models like Gemini-2.5pro can generate structurally rigorous analytical narratives but lack emphasis on critical evidence, resulting in incomplete reasoning chains. GPT-4o and OpenAI o3 produce more coherent and mature narratives, though they occasionally arrive at correct conclusions through relatively short chains of reasoning. In contrast, DeepSeek-R1 prioritizes maintaining the integrity of logical chains, but does not provide detailed reasoning content. This shows similarities with previous research findings, for example, ChatGPT-4.0 typically provides detailed program insights, while DeepSeek-R1 offers concise, structured responses but often lacks detailed elaboration (Prasad et al., 2025). These models exhibit different narrative styles and structural variations when generating output, and these differences have, to some extent, influenced the final scores. According to our rigorous evaluation criteria (for details, see Supplementary Table 2), a score of 4 indicates minor analytical oversights (e.g., failure to explicitly analyze contributing factors or exclude irrelevant variables) or incomplete logical reasoning. In contrast, a score of 5 demands a flawless, airtight chain of evidence—seamlessly linking the case history, physical and internal signs, microscopic findings, and final diagnosis. For example, in Case 28, the case details we provided explicitly included toxicology test results, stating that “No ethanol components were detected in Lin’s blood, and no common toxic (pharmacological) substances were detected in either the blood or gastric contents.” The analysis should explicitly rule out poisoning as a contributing cause of death; however, the output from Gemini-2.5pro lacks discussion or analysis of this aspect, resulting in an inference quality Score of 4, whereas other models all included analysis of this content. It is worth noting that these differing output styles also introduce a degree of subjectivity into the evaluation process, particularly regarding the inferential quality, where the level of inter-rater agreement was mostly moderate. Faced with models’ varying output preferences, different forensic experts may assign different weights to specific analytical dimensions, such as logical rigor, the completeness of exclusionary analysis, or the depth of pathophysiological descriptions. To some extent, these scoring discrepancies reflect a common practice in forensic work: In complex and challenging cases, expert panels often place varying emphasis on specific details within the chain of evidence. However, this phenomenon also highlights the subtle challenges involved in quantifying qualitative AI reasoning within the field of specialized medicine.

In this study, we observed that the four models also show significant differences (Figure 5) in their performance capabilities across different case types. In the task of inference quality, DeepSeek-R1 and Gemini-2.5pro showed statistically significant differences across injury-type (*p* = 0.016, *r*_rb_ = 0.52) and disease-type cases (*p* = 0.00012, *r*_rb_ = 0.49). In disease-type cases, OpenAI o3 also showed significant differences compared to Gemini-2.5 Pro (*p* = 0.04, *r*_rb_ = 0.36). Consistent with the above findings, no significant differences were shown in the task of conclusion accuracy. Interestingly, we show that across three case categories, the four models achieved the lowest proportion of perfect scores in injury and disease cases (GPT-4o: 11%, OpenAI o3: 15%, DeepSeek-R1: 26%), with Gemini-2.5pro not even receiving a single perfect score. This phenomenon may stem from our stringent evaluation criteria; since cases involving injury and disease inherently require a more complex web of evidence, the Gemini-2.5pro model may consequently overlook subtle logical connections. This phenomenon is consistent with the findings of the aforementioned studies, and research has shown that the quality and reliability of LLM outputs decline as the complexity of medical cases increases (Gumilar et al., 2024). However, in the task of conclusion accuracy, we showed an exception: DeepSeek-R1 achieved a perfect score in the injury and disease category (46%), showing that it outperformed its performance in the injury category alone (33%). We believe this is not an isolated phenomenon to be shown. In one study, ChatGPT-4 showed higher accuracy on questions deemed “difficult” compared to moderately challenging ones, and this trend persisted throughout a 30-day learning period (Devranoglu et al., 2024). This nonlinear pattern warrants further verification in subsequent research.

In exploratory research on local open-source LLMs, DeepSeek-R1:32b shows particularly noteworthy results. This model was selected for its massive parameter count (32 billion), which enables robust inference and deep semantic understanding. More importantly, this parameter model exhibits strong adaptability for local deployment and exceptional scalability, particularly suited for medium-to-high-end local computing environments. It is ideal for individuals or small-to-medium enterprises, making its evaluation more aligned with practical application scenarios. In this study, DeepSeek-R1 outperformed DeepSeek-R1:32b in the inference quality task (*p* = 0.0068*r*_rb_ = 0.49); no significant differences were noted in other tests or tasks (Figure 5). Of course, this phenomenon may be attributed to the research design and the source of the questions. All our case materials were translated from Chinese into English. Although the accuracy of the translations was meticulously verified, culturally specific forensic descriptors may lose precision during cross-linguistic transfer. For the DeepSeek series of products, which were trained on Chinese data, this undoubtedly represents a significant advantage. As Xu et al. (2025) showed in testing DeepSeek-R1 on complex ophthalmic reasoning tasks in China, its overall accuracy reached 0.862, ranking first in overall performance compared to other models such as Gemini 2.0 Pro and OpenAI O3-mini. Our research further confirms that the linguistic discrepancy between the initial training language and the language of user questions significantly impacts model performance (Ługowski et al., 2025).

We also conducted an extensive analysis of the content generated by DeepSeek-R1:32b. We found that, compared to existing general-purpose LLMs, the 32b output text is generally more concise, lacking detailed elaboration, and often resembling a structured outline rather than a complete forensic narrative. However, throughout this process, DeepSeek-R1:32b maintained the concise content and highly coherent reasoning logic chain characteristic of DeepSeek-R1 (Prasad et al., 2025). Its final output achieved a high score ratio of 93.3% in identification conclusions (Likert scores of 3 or higher, with the primary cause of death correctly identified). Although our test cases were limited, making this percentage potentially less statistically significant, the current performance of locally developed, open-source LLMs in forensic decision-making tasks represents a positive development for forensic practice. Research indicates that open-source LLMs can demonstrate potential comparable to proprietary LLMs, and in some cases, even outperform them (Sandmann et al., 2025). Deploying open-source models locally offers exceptional robustness (Kayaalp et al., 2025), providing practical value for organizations unable to utilize commercial API-based systems due to data privacy concerns, network independence requirements, or budget constraints. In radiology, researchers have demonstrated the potential for locally developed open-source models to replace top proprietary models (Li et al., 2025). Although locally deployed open-source models slightly lag behind top proprietary models in absolute performance, their configurations have demonstrated sufficient accuracy to support auxiliary roles, for example, preliminary case classification, drafting coroner report outlines, and cross-case evidence comparison, provided that outputs undergo expert validation.

It is important to emphasize that all five models exhibit varying degrees of “artificial hallucination,” which refers to the generation of outputs that appear plausible but are factually incorrect or fabricated (Hwang and Jeong, 2025). Although the incidence of such hallucinations is a cause for concern, some experts believe that hallucination is inherent to current generative AI systems because they are built upon the autoregressive design of LLMs (Lee et al., 2025). In our study, the factual incorrectness type exhibited the highest incidence of hallucinations. For example, in Case 4, Gemini-2.5 Pro misclassified the decedent’s antemortem epidermal abrasions on both thighs, knees and left elbow as “scald burns” caused by prolonged hot-water immersion. However, original histopathology confirmed dermal capillary congestion and vital hemorrhage, but no dermal collagen coagulative necrosis—the definitive criterion for excluding thermal scald injury in forensic pathology. This represents a serious factual misclassification and pathological misinterpretation, as the model proposed an entirely unsupported injury mechanism. This phenomenon poses a significant challenge in forensic practice (Christensen et al., 2025), particularly given the prevalence of automation bias, where individuals tend to over-trust AI outputs under the assumption of inherent algorithmic impartiality (Wingerter et al., 2025). In forensic applications, information accuracy is paramount, and reliance on AI-generated content must be exercised with extreme caution, lest AI outcomes impact our lives (Wang, 2025). Some attorneys have used AI-generated, fabricated legal precedents in litigation, resulting in court sanctions (Merken, 2023). From both a public and judicial perspective, such gaps in forensic reports can lead to ambiguity, prompting intense cross-examination by defense attorneys and potentially misleading the public or the jury. Such deficiencies often severely undermine judicial efficiency, necessitating supplementary testimony, lengthy back-and-forth exchanges, or even a comprehensive forensic re-examination.

In addition, we observed that model performance is highly dependent on the specific dimensions being evaluated, and there is a clear disconnect between the quality of reasoning and the accuracy of conclusions. In this study, we did not observe statistically significant differences among current, general-purpose LLMs in terms of conclusion accuracy. However, this statistical consistency merely indicates a lack of differences between models and does not imply the reliability of their reasoning conclusions. This phenomenon likely stems from the training datasets used: current general-purpose LLMs are primarily trained on comprehensive, open-domain datasets rather than specialized, forensic datasets, hence the absence of statistically detectable differences. Our research further indicates that performance gains and variations among LLMs are more pronounced at the “explanation layer” and “evidence weaving layer.” That is, differences in inference quality and the ability to integrate multi-source evidence are significantly more evident. These differences may stem from the unique training strategies and conceptual approaches adopted by different models. For example, the outstanding performance of DeepSeek-R1 and OpenAI o3 in reasoning is largely attributable to their deep optimization of the Chain-of-Thought (CoT) during the large-scale reinforcement learning (RL) phase (DeepSeek-AI et al., 2025). This architectural feature enables them to focus more on maintaining the integrity of the underlying chain of evidence in forensic analysis and to establish a robust medical reasoning framework (Wei et al., 2023). In contrast, GPT-4o relies heavily on reinforcement learning from human feedback (RLHF) (OpenAI et al., 2023), with training objectives that place a strong emphasis on the coherence and maturity of outputs, as well as human users’ reading preferences. While this enables it to demonstrate exceptional narrative capabilities, it occasionally resorts to “shortcut reasoning” in pursuit of efficiency, skipping over tedious intermediate exclusionary analyses (Ouyang et al., 2022; Turpin et al., 2023). The architecture of Gemini-2.5pro is designed with a focus on massive context windows and information throughput. While it excels at generating rigorously structured analytical frameworks, when processing highly complex forensic texts, it is prone to overlooking subtle logical connections within vast amounts of information if there are no strong alignment cues for key local evidence (Team G et al., 2024). However, the overall architectural design and training techniques of today’s leading large language models have reached a very high level of maturity. The widespread improvement in the capabilities of foundational models has effectively masked many underlying flaws in their derivations, leading to similar outcomes across models when handling routine tasks. This explains why, even though there are noticeable differences in the inference quality between models, the overall statistical effect remains relatively small.

The choice of LLM by users should be determined by their primary objective: when the research objective is to obtain a clear, reliable, and traceable chain of inference, particularly when assisting forensic experts in forming expert opinions or submitting evidence to judicial proceedings, subtle differences between models may substantially impact the integrity of conclusions and the accuracy of details. Such variations can lead to differing interpretations of specific facts. Users should opt for models like DeepSeek-R1 and OpenAI o3 that specialize in reasoning. Optimized through large-scale reinforcement learning (RL), these models are specifically designed to enhance computational capabilities during testing and leverage extended chain-of-thought (CoT) reasoning (DeepSeek-AI et al., 2025). This architecture inherently compels them to meticulously articulate intermediate logical steps before arriving at a final answer. Conversely, when the application requirement is merely to obtain a general direction of the conclusion, such as when the public, attorneys, or judges need only a preliminary understanding of possible causes of death, these top-tier models demonstrate broadly consistent performance in the accuracy of their final answers, within the scope of tasks covered by this study. However, the reliability of the results cannot be guaranteed, as our tests are statistically significant, and the model’s output still requires expert review. Therefore, a more reasonable approach is to position LLMs for auxiliary tasks such as evidence sorting, preliminary analysis, and report drafting, rather than replacing experts’ final judgments on key facts and legal standards. This also aligns with the majority of scholars’ views on the appropriate application of LLMs (Dinis-Oliveira and Azevedo, 2023; Aydogan et al., 2025). Conclusions generated by models must still undergo verification by professional forensic personnel to prevent judicial judgments from being misled by a chain of inferences that are plausible yet erroneous (Guleria et al., 2024).

## 5 Limitations and future prospects

As mentioned earlier, one limitation of this study is its reliance on a limited number of authentic Chinese forensic cases to evaluate the performance of LLMs. Due to differing legal systems and enforcement standards across countries, cases may exhibit significant variations between nations. Although we have endeavored to include cases of varying types and complexities, they cannot fully represent the complexity and diversity of international cases. Moreover, we currently lack sufficient sample sizes for testing, and this limited dataset may fail to fully capture rare yet critical edge cases or severe types of AI hallucinations. These general limitations on statistical power are particularly evident in our exploratory evaluation of a local, open-source model (DeepSeek-R1:32b), which was limited to a subset of 30 cases. Therefore, the hallucination rates reported in this paper should be regarded as preliminary estimates, rather than definitive prevalence figures. In future research, the development of a large-scale, cross-national, forensic database will be essential for verifying the general applicability of these forensic models across different jurisdictions. Moreover, it will be necessary to leverage more robust, local computing infrastructure to significantly expand the sample size, thereby providing a statistically conclusive assessment of whether privacy-preserving, local, large models can effectively replace closed-source, cloud-based models in confidential forensic settings.

At the same time, language limitations may impact model performance and yield different outcomes. To optimize the English reasoning capabilities of LLMs, we employed DeepL to machine-translate the original Chinese case documents. This approach indirectly dilutes the unique phrasing and complex sentences found in unstructured medical and autopsy records within the original texts. Future research will further evaluate performance differences in forensic decision-making across LLMs when processing different languages.

Furthermore, our approach is limited to standardized, zero-shot prompting strategies. While this design choice better aligns with real-world applications and reflects the model’s baseline performance, it also prevents the model from reaching its full reasoning potential. The absence of advanced prompting techniques, such as CoT or few-shot prompting, may prevent the model from handling highly complex evidentiary reasoning effectively. Future research should therefore explore prompt optimization techniques, as well as the impact of different prompts on reasoning quality and hallucination rates in forensic cases.

Finally, the evaluation in this study was conducted within a relatively short time window. Given the “black-box” nature of proprietary LLMs, as well as developers’ rapid and often unannounced iterative updates. Consequently, this study cannot verify the ability of LLMs to maintain diagnostic accuracy, reasoning consistency, and logical stability over an extended period. Before integrating generative AI into routine, high-stakes, forensic decision-making processes, longitudinal studies must be conducted, and continuous performance monitoring must be implemented.

## 6 Conclusion

Although the evaluation tasks here cover only a portion of the actual cause of death analysis tasks, our findings indicate that two highly correlated decision-making dimensions, inference quality and conclusion accuracy, demonstrate the tremendous potential of LLMs in assisting forensic decision-making. However, they currently lack the capacity for independent decision-making. Humans must enhance their ability to identify AI errors and understand algorithmic limitations, while assuming responsibility as the ultimate decision-makers (Gichoya et al., 2023; Ma and Su, 2025). We believe that by combining large multilingual case data with rigorous human oversight mechanisms, these models can show significant convenience and auxiliary support for forensic professionals and non-practitioners alike. Furthermore, this study demonstrates that the localized deployment of open-source LLMs represents a viable approach for integrating such auxiliary support tools into forensic practice applications. Due to the high reliance of forensic data on privacy protection and regulatory compliance, its practical application and local deployment still require careful consideration (Hefetz, 2023). Future multinational, multilingual forensic research must further validate whether the models can effectively translate into viable pathways for improving judicial decision-making quality and enhancing public health governance.

## Data Availability

The original contributions presented in the study are included in the article/Supplementary material, further inquiries can be directed to the corresponding authors.

## Footnotes

1
https://www.deepl.com/

2
https://chatgpt.com/?model=gpt-4o

3
https://chatgpt.com/?model=o3

4
https://gemini.google.com/app

5
https://chat.deepseek.com/

## References

[ref1] Ahuja K. Diddee H. Hada R. Ochieng M. Ramesh K. Jain P. MEGA: Multilingual evaluation of generative AI Bouamor H. Pino J. Bali K. Proceedings of the 2023 Conference on Empirical Methods in Natural Language Processing Singapore Association for Computational Linguistics. (2023). 4232–4267.

[ref2] Arena Leaderboard. (2025). Compare & Benchmark the Best Frontier AI Models. Available online at: https://arena.ai (Accessed May 28, 2025)

[ref3] Aydogan H. C. Yıkar B. Balandız H. Özsoy S. (2025). Assessing ChatGPT-4’s ability to generate forensic reports: a study of artificial intelligence in forensics. Egypt. J. Forensic Sci. 15:30. doi: 10.1186/s41935-025-00445-1

[ref4] Bonferroni C. (1936). Teoria statistica delle classi e calcolo delle probabilita, vol. 8. Florence, Italy: Pubblicazioni del R Istituto Superiore di Scienze Economiche e Commericiali di Firenze, 3–62.

[ref5] Cascella M. Montomoli J. Bellini V. Bignami E. (2023). Evaluating the feasibility of ChatGPT in healthcare: an analysis of multiple clinical and research scenarios. J. Med. Syst. 47:33. doi: 10.1007/s10916-023-01925-4, 36869927 PMC9985086

[ref6] Case Library of Judicial Administration (Legal Services). (2025). Available online at: https://alk.12348.gov.cn/ (Accessed September 14, 2025).

[ref7] Chiang W.-L. Zheng L. Sheng Y. Angelopoulos A. N. Li T. Li D. . (2024). Chatbot arena: an open platform for evaluating LLMs by human preference. doi: 10.48550/arXiv.2403.04132

[ref8] Cho S. H. Kim D. Kwon H. C. Kim M. (2024). Exploring the potential of large language models for author profiling tasks in digital text forensics. Forensic Sci. Int. Digit. Investig. 50:301814. doi: 10.1016/j.fsidi.2024.301814, 38826717

[ref9] Christensen J. Hansen J. M. Wilson P. (2025). Understanding the role and impact of generative artificial intelligence (AI) hallucination within consumers’ tourism decision-making processes. Curr. Issue Tour. 28, 545–560. doi: 10.1080/13683500.2023.2300032

[ref10] Cooper G. (2023). Examining science education in ChatGPT: an exploratory study of generative artificial intelligence. J. Sci. Educ. Technol. 32, 444–452. doi: 10.1007/s10956-023-10039-y

[ref11] DeepSeek-AI Guo D. Yang D. Zhang H. Song J. Wang P. . (2025). DeepSeek-R1: incentivizing reasoning capability in LLMs via reinforcement learning. arXiv. doi: 10.48550/ARXIV.2501.12948

[ref12] Devranoglu B. Gurbuz T. Gokmen O. (2024). Chatgpt’s efficacy in queries regarding polycystic ovary syndrome and treatment strategies for women experiencing infertility. Diagnostics 14:1082. doi: 10.3390/diagnostics14111082, 38893609 PMC11172366

[ref13] Dinis-Oliveira R. J. Azevedo R. M. S. (2023). ChatGPT in forensic sciences: a new pandora’s box with advantages and challenges to pay attention. Forensic Sci Res 8, 275–279. doi: 10.1093/fsr/owad039, 38405625 PMC10894065

[ref14] Feuerriegel S. Hartmann J. Janiesch C. Zschech P. (2024). Generative AI. Bus. Inf. Syst. Eng. 66, 111–126. doi: 10.1007/s12599-023-00834-7

[ref15] Gibney E. (2025). China’s cheap, open AI model DeepSeek thrills scientists. Nature 638, 13–14. doi: 10.1038/d41586-025-00229-6, 39849139

[ref16] Gichoya J. W. Thomas K. Celi L. A. Safdar N. Banerjee I. Banja J. D. . (2023). AI pitfalls and what not to do: mitigating bias in AI. Br. J. Radiol. 96:20230023. doi: 10.1259/bjr.20230023, 37698583 PMC10546443

[ref17] Google. Gemini 2.5: Our most intelligent models are getting even better. Google (2025). Available online at: https://blog.google/technology/google-deepmind/google-gemini-updates-io-2025/ (Accessed May 30, 2025).

[ref18] Guleria A. Krishan K. Sharma V. Kanchan T. (2024). ChatGPT: forensic, legal, and ethical issues. Med. Sci. Law 64, 150–156. doi: 10.1177/00258024231191829, 37528607

[ref19] Gumilar K. E. Indraprasta B. R. Faridzi A. S. Wibowo B. M. Herlambang A. Rahestyningtyas E. . (2024). Assessment of large language models (LLMs) in decision-making support for gynecologic oncology. Comput. Struct. Biotechnol. J. 23, 4019–4026. doi: 10.1016/j.csbj.2024.10.050, 39610903 PMC11603009

[ref20] Hefetz I. (2023). Mapping AI-ethics’ dilemmas in forensic case work: to trust AI or not? Forensic Sci. Int. 350:111807. doi: 10.1016/j.forsciint.2023.111807, 37567040

[ref21] Hwang Y. Jeong S.-H. (2025). Generative artificial intelligence and misinformation acceptance: an experimental test of the effect of forewarning about artificial intelligence hallucination. Cyberpsychol. Behav. Soc. Netw. 28, 284–289. doi: 10.1089/cyber.2024.0407, 39992238

[ref22] ICD-11 for Mortality and Morbidity Statistics. (2025). Available online at: https://icd.who.int/browse/2025-01/mms/en#435227771 (Accessed August 28, 2025).

[ref23] Inoue K. Abe S. Fukunaga T. (2023). Current state of cause of death determinations in Japan and the need to list the precise underlying cause of death. Med. Sci. Law 63, 114–119. doi: 10.1177/00258024221102203, 35585706

[ref24] Kayaalp M. E. Prill R. Sezgin E. A. Cong T. Królikowska A. Hirschmann M. T. (2025). DeepSeek versus ChatGPT: multimodal artificial intelligence revolutionizing scientific discovery. From language editing to autonomous content generation—redefining innovation in research and practice. Knee Surg. Sports Traumatol. Arthrosc. 33, 1553–1556. doi: 10.1002/ksa.12628, 39936363

[ref25] Kim K. J. Lee C. H. Bae S. E. Choi J. H. Kang W. (2025). Digital forensics in law enforcement: a case study of LLM-driven evidence analysis. Forensic Sci. Int. Digit. Investig. 54:301939. doi: 10.1016/j.fsidi.2025.301939, 38826717

[ref26] Landis J. R. Koch G. G. (1977). The measurement of observer agreement for categorical data. Biometrics 33, 159–174. doi: 10.2307/2529310, 843571

[ref27] Lee C. Kim J. Lim J. S. Shin D. (2025). Generative AI risks and resilience: how users adapt to hallucination and privacy challenges. Telemat. Inform. Rep. 19:100221. doi: 10.1016/j.teler.2025.100221

[ref28] Li D. Gupta K. Bhaduri M. Sathiadoss P. Bhatnagar S. Chong J. (2025). Comparative diagnostic accuracy of GPT-4o and LLaMA 3-70b: proprietary vs. open-source large language models in radiology. Clin. Imaging 118:110382. doi: 10.1016/j.clinimag.2024.110382, 39740646

[ref29] Ługowski F. Babińska J. Ludwin A. Stanirowski P. J. (2025). Comparative analysis of ChatGPT 3.5 and ChatGPT 4 obstetric and gynecological knowledge. Sci. Rep. 15:21133. doi: 10.1038/s41598-025-08424-1, 40596684 PMC12216761

[ref30] Ma H. Su M. (2025). Whom to sue? Liability of unaccountability in AI decisions. Organ. Dyn. 54:101123. doi: 10.1016/j.orgdyn.2024.101123

[ref31] Madea B. Prangenberg J. Doberentz E. (2022). Feststellung der todesursache. Rechtsmedizin 32, 221–236. doi: 10.1007/s00194-021-00548-8

[ref32] Massenon R. Gambo I. Khan J. A. Agbonkhese C. Alwadain A. (2025). “My AI is lying to me”: user-reported LLM hallucinations in AI mobile apps reviews. Sci. Rep. 15:30397. doi: 10.1038/s41598-025-15416-8, 40830185 PMC12365265

[ref33] Meilia P. D. I. Freeman M. D. Herkutanto Zeegers M. P. (2020). A review of causal inference in forensic medicine. Forensic Sci. Med. Pathol. 16, 313–320. doi: 10.1007/s12024-020-00220-9, 32157581 PMC7245596

[ref34] Merken S. New York Lawyers Sanctioned for using fake ChatGPT Cases in legal Brief. Reuters (2023) Available online at: https://www.reuters.com/legal/new-york-lawyers-sanctioned-using-fake-chatgpt-cases-legal-brief-2023-06-22/ (Accessed August 20, 2025).

[ref35] Mesko B. (2023). The ChatGPT (generative artificial intelligence) revolution has made artificial intelligence approachable for medical professionals. J. Med. Internet Res. 25:e48392. doi: 10.2196/48392, 37347508 PMC10337400

[ref36] Michelet G. Breitinger F. (2024). ChatGPT, llama, can you write my report? An experiment on assisted digital forensics reports written using (local) large language models. Forensic Sci. Int. Digit. Investig. 48:301683. doi: 10.1016/j.fsidi.2023.301683, 38826717

[ref37] Obafunwa J. O. Ajayi O. Okoye M. I. (2018). Medical evidence and proof of cause of death in nigerian courts. Med. Sci. Law 58, 122–134. doi: 10.1177/0025802418754576, 29381106

[ref38] Ollama. (2025). Available online at: https://ollama.com (Accessed July 8, 2025).

[ref39] OpenAI Achiam J. Adler S. Agarwal S. Ahmad L. Akkaya I. . (2023). GPT-4 technical report. arXiv. doi: 10.48550/ARXIV.2303.08774

[ref40] OpenAI research. (2025). Available online at: https://openai.com/research/index/ (Accessed May 30, 2025)

[ref41] Ouyang L. Wu J. Jiang X. Almeida D. Wainwright C. L. Mishkin P. . (2022). Training language models to follow instructions with human feedback. arXiv. doi: 10.48550/ARXIV.2203.02155

[ref42] Patel K. Say S. Leng D. Khut S. Duong S. Ly C. . (2025). Use of the international classification of diseases to perinatal mortality (ICD-PM) with verbal autopsy to determine the causes of stillbirths and neonatal deaths in rural Cambodia: a population-based, prospective, cohort study. Lancet Reg. Health West. Pacific 60:101626. doi: 10.1016/j.lanwpc.2025.101626, 40697534 PMC12282259

[ref43] Petroni G. Alaimo S. Mandarelli G. Catanesi R. Niolu C. Siracusano A. . (2025). A case study of forensic psychiatry experts’ reports analysis through large language models. Int. J. Law Psychiatry 102:102122. doi: 10.1016/j.ijlp.2025.102122, 40516226

[ref44] Prasad S. Langlie J. Pasick L. Chen R. Franzmann E. (2025). Evaluating advanced AI reasoning models: ChatGPT-4.0 and DeepSeek-R1 diagnostic performance in otolaryngology: a comparative analysis. Am. J. Otolaryngol. 46:104667. doi: 10.1016/j.amjoto.2025.104667 40367837

[ref45] Sandmann S. Hegselmann S. Fujarski M. Bickmann L. Wild B. Eils R. . (2025). Benchmark evaluation of DeepSeek large language models in clinical decision-making. Nat. Med. 31, 2546–2549. doi: 10.1038/s41591-025-03727-2, 40267970 PMC12353792

[ref46] Sandmann S. Riepenhausen S. Plagwitz L. Varghese J. (2024). Systematic analysis of ChatGPT, google search and llama 2 for clinical decision support tasks. Nat. Commun. 15:2050. doi: 10.1038/s41467-024-46411-8, 38448475 PMC10917796

[ref47] Scott R. (2017). The murder trial of gerard Baden-clay: admissibility of expert opinion evidence of injuries and cause of death. J. Law Med. 25, 166–204. doi: 10.1080/13218719.2017.1379113, 29978631

[ref48] Team G Georgiev P. Lei V. I. Burnell R. Bai L. Gulati A. . (2024). Gemini 1.5: unlocking multimodal understanding across millions of tokens of context. arXiv. doi: 10.48550/arXiv.2403.05530

[ref49] Temsah A. Alhasan K. Altamimi I. Jamal A. Al-Eyadhy A. Malki K. H. . (2025). DeepSeek in healthcare: revealing opportunities and steering challenges of a new open-source artificial intelligence frontier. Cureus 17:e79221. doi: 10.7759/cureus.79221, 39974299 PMC11836063

[ref50] Turpin M. Michael J. Perez E. Bowman S. R. (2023). Language models don’t always say what they think: unfaithful explanations in chain-of-thought prompting. arXiv. doi: 10.48550/arXiv.2305.04388

[ref51] Tyagi S. Gong Y. Karabiyik U. (2025). Forensic analysis and privacy implications of LLM mobile apps: a case study of ChatGPT, copilot, and gemini. Forensic Sci. Int. 54:301974. doi: 10.1016/j.fsidi.2025.301974, 38826717

[ref52] Wang Q. (2025). Beyond pandora’s box: vast potential with significant challenges. Forensic Sciences Research 10:owae017. doi: 10.1093/fsr/owae017, 39990699 PMC11839503

[ref53] Wang L. Chen X. Deng X. Wen H. You M. Liu W. . (2024). Prompt engineering in consistency and reliability with the evidence-based guideline for LLMs. npj Digit Med 7:41. doi: 10.1038/s41746-024-01029-4, 38378899 PMC10879172

[ref54] Wei J. Wang X. Schuurmans D. Bosma M. Ichter B. Xia F. . (2023). Chain-of-thought prompting elicits reasoning in large language models. arXiv. doi: 10.48550/arXiv.2201.11903

[ref55] Wickramasekara A. Breitinger F. Scanlon M. (2025). Exploring the potential of large language models for improving digital forensic investigation efficiency. Forensic Sci. Int. Digit. Investig. 52:301859. doi: 10.1016/j.fsidi.2024.301859, 38826717

[ref56] Wingerter T. L. Straub T. Schweitzer S. (2025). Mitigating automation bias in generative AI through nudges: a cognitive reflection test study. Procedia Comput. Sci. 270, 2106–2114. doi: 10.1016/j.procs.2025.09.331

[ref57] Xu P. Wu Y. Jin K. Chen X. He M. Shi D. (2025). DeepSeek-R1 outperforms gemini 2.0 pro, OpenAI o1, and o3-mini in bilingual complex ophthalmology reasoning. Adv. Ophthalmol. Pract. Res. 5, 189–195. doi: 10.1016/j.aopr.2025.05.001, 40678192 PMC12269606

[ref58] Yu Z. Fang L. Ding Y. Shen Y. Xu L. Cai Y. . (2025). Evaluating large language models for information extraction from gastroscopy and colonoscopy reports through multi-strategy prompting. J. Biomed. Inform. 168:104844. doi: 10.1016/j.jbi.2025.104844, 40505790

