# Supplementary material for: Can large language models serve as consultants for forensic cause of death analysis? A multidimensional evaluation
Source: Front Artif Intell. 2026 Jul 28;9:1752043. doi: 10.3389/frai.2026.1752043 (PMC13457641; doi:10.3389/frai.2026.1752043)
Supplement: Supplementary file 1 [file Table_3.DOCX]

**Supplementary Table 1. Friedman test results comparing overall and case-type-specific performance among the four general-purpose LLMs.**

| **Dimension** | **Case Category** | **Sample Size (N)** | **df** | **Friedman X^2^** | **P_value** | **Kendall's W** |
| --- | --- | --- | --- | --- | --- | --- |
| **inference quality** | All Cases | 118 | 3 | 27.163 | < 0.001 | 0.077 |
|  | Case Type 1 (disease) | 58 | 3 | 10.6 | 0.014101 | 0.061 |
|  | Case Type 2 (injury) | 33 | 3 | 13.277 | 0.0040737 | 0.134 |
|  | Case Type 3 (disease & injury) | 27 | 3 | 5.335 | 0.14886 | 0.066 |
| **conclusion accuracy** | All Cases | 118 | 3 | 3.803 | 0.28351 | 0.011 |
|  | Case Type 1 (disease) | 58 | 3 | 2.156 | 0.54062 | 0.012 |
|  | Case Type 2 (injury) | 33 | 3 | 5.139 | 0.1619 | 0.052 |
|  | Case Type 3 (disease & injury) | 27 | 3 | 2.343 | 0.50436 | 0.029 |

**Supplementary Table 2. The 5-Point Likert Scale Rubric for Evaluating LLM Performance in Forensic Cause-of-Death Analysis**

| **Score** | **Inference quality** | **Conclusion accuracy** |
| --- | --- | --- |
| **1** | Severe Omissions & Broken Logic: Missing core elements (element ①, or ≥2 elements) and substantially incomplete reasoning logic involving critical injury/disease signs. | Completely Incorrect: Incorrect identification of the primary cause of death, with all contributing factors either unmentioned or completely misdiagnosed. |
| **2** | Severe Omissions & Partial Logic: Missing core elements (element ①, or ≥2 elements) and incomplete reasoning logic involving only non-critical injury/disease signs. | Primary Cause Incorrect: Incorrect identification of the primary cause of death, but contributing factors are correctly or partially correctly identified. |
| **3** | Partial Omissions & Broken Logic: Missing one secondary element (element ② or ③) and incomplete reasoning logic. | Primary Cause Correct: Correct identification of the primary cause of death, but all contributing factors are unmentioned or completely misdiagnosed. |
| **4** | Partial Omissions or Logical Flaws: Missing one secondary element (element ② or ③); OR slightly incomplete reasoning logic; OR minor factual errors during the analysis. | Primary Cause Correct (Minor Flaws): Correct identification of the primary cause of death, with contributing factors only partially mentioned or containing minor errors.  (Note: If the case contains only a single contributing factor and it is omitted or misdiagnosed, the score is downgraded to 3). |
| **5** | Completely Inference: No omissions in content elements and a completely rigorous and comprehensive logical chain. | Completely Accurate: The primary cause of death and all contributing elements are explicitly mentioned and correctly diagnosed. |

Evaluation Guidelines for Inference quality:
**A.** Content Elements: ① Core Element: Explicit analysis of the primary cause of death. ② Secondary Element: Analysis of contributing factors (e.g., underlying diseases, precipitating factors, or auxiliary causes of death). ③ Exclusion Element: Explicit exclusion of unrelated factors (e.g., adequately ruling out suspected poisoning, non-lethal diseases, or non-lethal injuries).

**B.** Standardized Reasoning Logic Chain: A complete logical sequence should seamlessly connect the following evidence nodes: Case History/Medical History → Macroscopic Autopsy Signs (External) → Internal Autopsy Signs → Microscopic Findings → Final Diagnosis.

**C.** Reference Analytical Approach: Gain an understanding of the situation through case details and medical records → Autopsy and pathological examination determine the cause of death → Exclude non-lethal factors → Synthesize findings → Formulate the final conclusion.

**Supplementary Table 3. Performance and results of statistical tests per task for GPT-4o, OpenAI o3, DeepSeek-R1 and Gemini-2.5pro.**

Performance of GPT-4o, OpenAI o3, DeepSeek-R1, and Gemini-2.5pro is measured using median scores and interquartile ranges. Two- sided Wilcoxon signed-rank test was applied for statistical testing (adjusted with Bonferroni correction for multiple testing considering n=6 tests for process, n=6 tests for conclusion).

| **Dimension** | **Comparison_Model_1** | **Comparison_Model_2** | **Model 1**  **[median;IQR]** | **Model 2**  **[median;IQR]** | **Hl_estimate** | **P values** | **Adjusted_P** | **95%CI** | **Effect**  **_size_r** | **Statistic_V** |
| --- | --- | --- | --- | --- | --- | --- | --- | --- | --- | --- |
| **inference quality** | Deepseek-R1 | Gemini-2.5pro | 4.50 [4.00; 5.00] | 4.00 [4.00; 4.50] | 7.499225e-01 | 0.00000050 | 2.989979e-06 | [4.999316e-01; 7.500158e-01] | 0.46276870 | 2954.0 |
|  | Deepseek-R1 | GPT-4o | 4.50 [4.00; 5.00] | 4.00 [4.00; 4.50] | 2.500154e-01 | 0.00251311 | 1.507866e-02 | [1.951823e-05; 7.499753e-01] | 0.27817541 | 2842.0 |
|  | Deepseek-R1 | OpenAI o3 | 4.50 [4.00; 5.00] | 4.00 [4.00; 5.00] | 2.499246e-01 | 0.05297409 | 3.178446e-01 | [-6.906065e-05; 2.500541e-01] | 0.17814334 | 2210.5 |
|  | Gemini-2.5pro | GPT-4o | 4.00 [4.00; 4.50] | 4.00 [4.00; 4.50] | -2.499717e-01 | 0.07578188 | 4.546913e-01 | [-4.999765e-01; 3.280214e-05] | 0.16346671 | 1327.0 |
|  | OpenAI o3 | Gemini-2.5pro | 4.00 [4.00; 5.00] | 4.00 [4.00; 4.50] | 4.999439e-01 | 0.00059657 | 3.579442e-03 | [2.499540e-01; 7.499623e-01] | 0.31604868 | 2375.5 |
|  | OpenAI o3 | GPT-4o | 4.00 [4.00; 5.00] | 4.00 [4.00; 4.50] | 2.499912e-01 | 0.07134103 | 4.280462e-01 | [-6.775187e-06; 2.500070e-01] | 0.16600727 | 2180.5 |

**Supplementary Table 4. Performance and results of statistical tests per task for GPT-4o, DeepSeek-R1 and DeepSeek-R1:32b.**

Performance of GPT-4o, OpenAI o3, DeepSeek-R1, and Gemini-2.5pro is measured using median scores and interquartile ranges. One- sided Wilcoxon signed-rank test (alternative = greater) was applied for statistical testing (adjusted with Bonferroni correction for multiple testing considering n=2 tests for process, n=2 tests for conclusion).

| **Dimension** | **Comparison_Model_1** | **Comparison_Model_2** | **Model 1**  **[median;IQR]** | **Model 2**  **[median;IQR]** | **Hl_estimate** | **P values** | **Adjusted_P** | **95%CI** | **Effect_size_r** | **Statistic_V** |
| --- | --- | --- | --- | --- | --- | --- | --- | --- | --- | --- |
| **inference quality** | Deepseek-R1 | Deepseek-R1:32b | 4.25 [4.00; 5.00] | 4.00 [3.62; 4.50] | 7.499403e-01 | 0.00344283 | 0.00688567 | [0.2500133; Inf] | 0.49337454 | 161.0 |
|  | GPT-4o | Deepseek-R1:32b | 4.00 [4.00; 4.00] | 4.00 [3.62; 4.50] | 7.542620e-06 | 0.29753825 | 0.59507650 | [-0.2499591; Inf] | 0.09703708 | 131.0 |
| **conclusion accuracy** | Deepseek-R1 | Deepseek-R1:32b | 4.75 [4.00; 5.00] | 4.50 [3.25; 5.00] | 4.239802e-05 | 0.5 | 1.00 | [-0.4999551; Inf] | 0.00000000 | 95.5 |
|  | GPT-4o | Deepseek-R1:32b | 4.50 [4.00; 5.00] | 4.50 [3.25; 5.00] | -8.531278e-05 | 0.66500883 | 1.00 | [-0.7499412; Inf] | 0.07780805 | 114.0 |

**Supplementary Table 5. Performance and results of statistical tests per task** **in injury-type cases (n = 33) for GPT-4o, OpenAI o3, DeepSeek-R1 and Gemini-2.5pro.** Performance in injury-type cases of GPT-4o, OpenAI o3, DeepSeek-R1, and Gemini-2.5pro is measured using median scores and interquartile ranges. One- sided Wilcoxon signed-rank test (alternative = greater) was applied for statistical testing in injury-type cases (adjusted with Bonferroni correction for multiple testing considering n=6 tests for process, n=6 tests for conclusion).

| **Dimension** | **Comparison_Model_1** | **Comparison_Model_2** | **Model 1**  **[median;IQR]** | **Model 2**  **[median;IQR]** | **Hl_estimate** | **P values** | **Adjusted_P** | **95%CI** | **Effect_size_r** | **Statistic_V** |
| --- | --- | --- | --- | --- | --- | --- | --- | --- | --- | --- |
| **inference quality** | Deepseek-R1 | Gemini-2.5pro | 4.50 [4.00; 5.00] | 4.00 [4.00; 4.50] | 7.499991e-01 | 0.00267421 | 0.01604528 | [4.999934e-01; Inf] | 0.52273759 | 228.5 |
|  | Deepseek-R1 | GPT-4o | 4.50 [4.00; 5.00] | 4.50 [4.00; 4.50] | 4.999896e-01 | 0.07406343 | 0.44438061 | [-4.080612e-06; Inf] | 0.31094122 | 215.5 |
|  | Deepseek-R1 | OpenAI o3 | 4.50 [4.00; 5.00] | 4.50 [4.00; 5.00] | 5.685220e-05 | 0.22171899 | 1.00 | [-2.499107e-01; Inf] | 0.21271767 | 150.0 |
|  | GPT-4o | Gemini-2.5pro | 4.50 [4.00; 4.50] | 4.00 [4.00; 4.50] | 2.500330e-01 | 0.07591245 | 0.45547467 | [-2.177374e-05; Inf] | 0.30897249 | 184.0 |
|  | OpenAI o3 | Gemini-2.5pro | 4.50 [4.00; 5.00] | 4.00 [4.00; 4.50] | 5.000158e-01 | 0.01026108 | 0.06156647 | [2.499801e-01; Inf] | 0.44684076 | 197.0 |
|  | OpenAI o3 | GPT-4o | 4.50 [4.00; 5.00] | 4.50 [4.00; 4.50] | 2.499912e-01 | 0.16205144 | 0.97230865 | [-1.527990e-05; Inf] | 0.24339629 | 170.0 |

**Supplementary Table 6. Performance and results of statistical tests per task in disease-type cases (n = 58) for GPT-4o, OpenAI o3, DeepSeek-R1 and Gemini-2.5pro.** Performance in disease-type cases of GPT-4o, OpenAI o3, DeepSeek-R1, and Gemini-2.5pro is measured using median scores and interquartile ranges. One- sided Wilcoxon signed-rank test (alternative = greater) was applied for statistical testing in disease-type cases (adjusted with Bonferroni correction for multiple testing considering n=6 tests for process, n=6 tests for conclusion).

| **Dimension** | **Comparison_Model_1** | **Comparison_Model_2** | **Model 1**  **[median;IQR]** | **Model 2**  **[median;IQR]** | **Hl_estimate** | **P values** | **Adjusted_P** | **95%CI** | **Effect_size_r** | **Statistic_V** |
| --- | --- | --- | --- | --- | --- | --- | --- | --- | --- | --- |
| **inference quality** | Deepseek-R1 | Gemini-2.5pro | 4.25 [4.00; 5.00] | 4.00 [4.00; 4.50] | 5.000472e-01 | 0.00020619 | 0.001237141 | [2.500716e-01; Inf] | 0.48731875 | 729.0 |
|  | Deepseek-R1 | GPT-4o | 4.25 [4.00; 5.00] | 4.00 [4.00; 4.50] | 2.500446e-01 | 0.01117818 | 0.067069097 | [3.041122e-05; Inf] | 0.33313475 | 687.0 |
|  | Deepseek-R1 | OpenAI o3 | 4.25 [4.00; 5.00] | 4.00 [4.00; 5.00] | 2.499494e-01 | 0.14302835 | 0.858170109 | [-1.386863e-05; Inf] | 0.19231223 | 465.5 |
|  | GPT-4o | Gemini-2.5pro | 4.00 [4.00; 4.50] | 4.00 [4.00; 4.50] | 2.499750e-01 | 0.10225586 | 0.613535146 | [-4.974451e-05; Inf] | 0.21455658 | 526.5 |
|  | OpenAI o3 | Gemini-2.5pro | 4.00 [4.00; 5.00] | 4.00 [4.00; 4.50] | 5.000101e-01 | 0.00668297 | 0.040097798 | [2.499311e-01; Inf] | 0.35613490 | 539.0 |
|  | OpenAI o3 | GPT-4o | 4.00 [4.00; 5.00] | 4.00 [4.00; 4.50] | 2.499761e-01 | 0.08810989 | 0.528659349 | [-4.029152e-05; Inf] | 0.22393701 | 440.0 |

**Supplementary** **Table 7. Performance and results of statistical tests per task in injury and disease-type cases (n = 27) for GPT-4o, OpenAI o3, DeepSeek-R1 and Gemini-2.5pro.** Performance in injury and disease-type cases of GPT-4o, OpenAI o3, DeepSeek-R1, and Gemini-2.5pro is measured using median scores and interquartile ranges. One- sided Wilcoxon signed-rank test (alternative = greater) was applied for statistical testing in injury and disease-type cases (adjusted with Bonferroni correction for multiple testing considering n=6 tests for process, n=6 tests for conclusion).

| **Dimension** | **Comparison_Model_1** | **Comparison_Model_2** | **Model 1**  **[median;IQR]** | **Model 2**  **[median;IQR]** | **Hl_estimate** | **P values** | **Adjusted_P** | **95%CI** | **Effect_size_r** | **Statistic_V** |
| --- | --- | --- | --- | --- | --- | --- | --- | --- | --- | --- |
| **inference quality** | Deepseek-R1 | Gemini-2.5pro | 4.00 [4.00; 4.75] | 4.00 [3.50; 4.00] | 7.499605e-01 | 0.01263030 | 0.07578179 | [2.499398e-01; Inf] | 0.47997569 | 164.5 |
|  | Deepseek-R1 | GPT-4o | 4.00 [4.00; 4.75] | 4.00 [3.25; 4.50] | 5.000000e-01 | 0.07411215 | 0.44467287 | [-2.393343e-05; Inf] | 0.34370054 | 171.0 |
|  | Deepseek-R1 | OpenAI o3 | 4.00 [4.00; 4.75] | 4.00 [3.50; 4.25] | 2.500109e-01 | 0.06095416 | 0.36572498 | [-1.878684e-05; Inf] | 0.36061833 | 188.5 |
|  | GPT-4o | Gemini-2.5pro | 4.00 [3.25; 4.50] | 4.00 [3.50; 4.00] | 1.335155e-05 | 0.39501468 | 1.00 | [-4.999514e-01; Inf] | 0.16369005 | 92.0 |
|  | OpenAI o3 | Gemini-2.5pro | 4.00 [3.50; 4.25] | 4.00 [3.50; 4.00] | 2.929591e-05 | 0.18529765 | 1.00 | [-4.336320e-05; Inf] | 0.25492299 | 141.0 |
|  | OpenAI o3 | GPT-4o | 4.00 [3.50; 4.25] | 4.00 [3.25; 4.50] | 7.895427e-05 | 0.26468711 | 1.00 | [-2.499678e-01; Inf] | 0.21465521 | 172.0 |
